# Efficient electron-induced removal of oxalate ions and formation of copper nanoparticles from copper(II) oxalate precursor layers

**DOI:** 10.3762/bjnano.7.77

**Published:** 2016-06-13

**Authors:** Kai Rückriem, Sarah Grotheer, Henning Vieker, Paul Penner, André Beyer, Armin Gölzhäuser, Petra Swiderek

**Affiliations:** 1 University of Bremen, Institute for Applied and Physical Chemistry, Fachbereich 2 (Chemie/Biologie), Leobener Straße / NW 2, Postfach 330440, D-28334 Bremen, Germany; 2 Physics of Supramolecular Systems, Bielefeld University, D-33615 Bielefeld, Germany

**Keywords:** copper(II) oxalate, electron-induced reactions, layer-by-layer deposition, nanoparticle formation, thin film

## Abstract

Copper(II) oxalate grown on carboxy-terminated self-assembled monolayers (SAM) using a step-by-step approach was used as precursor for the electron-induced synthesis of surface-supported copper nanoparticles. The precursor material was deposited by dipping the surfaces alternately in ethanolic solutions of copper(II) acetate and oxalic acid with intermediate thorough rinsing steps. The deposition of copper(II) oxalate and the efficient electron-induced removal of the oxalate ions was monitored by reflection absorption infrared spectroscopy (RAIRS). Helium ion microscopy (HIM) reveals the formation of spherical nanoparticles with well-defined size and X-ray photoelectron spectroscopy (XPS) confirms their metallic nature. Continued irradiation after depletion of oxalate does not lead to further particle growth giving evidence that nanoparticle formation is primarily controlled by the available amount of precursor.

## Introduction

Electron-induced chemistry is a versatile approach to the fabrication of nanoscale materials and devices. In fact, depending on the electron source used in such processes, different types of nanostructures are accessible. Using a tightly focused beam, structures of arbitrary shape with dimensions in the nanometer regime can be directly written on surfaces. In such focused electron beam induced deposition (FEBID) [1–2] solid materials are produced on surfaces through decomposition of volatile precursor compounds under the electron beam [1,3–4]. As an alternative to deposition from the gas phase, FEBID has recently also been performed in micrometer-thin films of molten metal salts [5] or in aqueous precursor solutions [6–8].

In contrast, divergent lower-energy electron beams process surfaces on macroscopic length scales. In this case, patterns can be imprinted onto a surface by electron exposure through a mask [9–10]. Such patterns themselves often consist of smaller structures, namely, when electron exposure leads to formation of nanoparticles (NPs) in the irradiated surface area. Hierarchical surface patterns are thus accessible. In fact, the formation of metal NPs under electron irradiation has been observed in diverse precursor materials such as solid [11–13] and molten [5] metal salts or their aqueous solutions [6,8] as well as in polymers loaded with metal salts [14–17], metal-organic frameworks [18] and spin-coated assemblies [19].

Independent of the width and energy of the electron beam, the purity of the deposited material [18,20] and the control of the size of the nanostructures pose challenges [1,11–13,18,20]. For example, when metals are deposited from the gas phase by FEBID, the organic ligands that provide the metal organic precursors with sufficient volatility are often not fully decomposed. In consequence, organic residues become embedded in the deposit [1,20]. Pure metal NPs are more easily accessible through electron exposure of solid metal salts containing small anions [11–13]. However, the NPs are often formed with a wide size distribution, which partly relates to the fact that precursor preparation by drying solutions at a surface usually does not yield a layer with homogeneous thickness but rather aggregates of small crystallites [12–13]. In contrast, when polymer matrices deposited by spin-coating and containing ionic Au were irradiated [14–15], the NP size could be tuned by controlling the layer thickness [15]. Washing and pyrolysis of the molecular residues was then necessary to obtain pure metal NPs, the latter unfortunately inducing post-irradiation particle growth ascribed to Ostwald ripening and therefore deteriorating the monodispersity [15]. As an alternative, a homogeneous precursor distribution on the surface was achieved by using liquid precursor materials, i.e., molten salts [5] or solutions of the precursors [6–8]. The latter processes are more demanding in terms of instrumentation as they require heating stages [5] or liquid cells [6–8], respectively. Furthermore, the applied electron energies are dictated by the layer thickness to be penetrated.

The examples discussed so far suggest that metal-containing self-assembled layers with well-defined thickness are advantageous when used as precursors for electron-induced nanostructure formation. For instance, a rather homogeneous distribution of monodisperse NPs has been achieved by electron irradiation and subsequent washing of well-organized silver(I) dodecanethiolate layers [19]. Also, self-assembled layers can be prepared by simple wet-chemical dipping processes [21–22] or high-throughput spray applications [23]. Layer-by-layer deposition processes employing repeated dipping steps lead to materials of well-defined thickness, an example being surface-mounted metal-organic frameworks (SurMOFs) [24]. In such materials, the metal ion surface density can be precisely controlled [25] which, in turn, should be an important factor in tuning the size of nanostructures formed under electron exposure. The formation of crystalline Cu NPs by electron-induced reduction of the Cu^2+^ ions in the framework has been observed in the corresponding bulk MOF material HKUST-1 [18]. This process should equally occur in the corresponding SurMOF making it an apparently interesting precursor material. However, the NPs produced by exposing HKUST-1 to electrons were embedded in an ill-defined carbon matrix [18] calling again for further purification steps.

The aim of the present study was to demonstrate that copper(II) oxalate is a material that has particularly favorable properties as a precursor for electron-induced nanoparticle formation at surfaces. Surface layers of this compound can be prepared with well-defined thickness using a recently established layer-by-layer deposition procedure [26]. Similar to the self-assembled layers with well-defined numbers of binding sites for metal ions described above, surface-grown layers of copper(II) oxalate contain a well-defined surface density of metal ions, which is a prerequisite to tune NP sizes or surface density in the subsequent irradiation step. Here we show that surface-grown layers of copper(II) oxalate are, in fact, transformed to pure Cu NPs by low-energy electron irradiation at room temperature while the oxalate ions are completely removed through the same electron-induced process that yields the NPs. This makes post-irradiation steps obsolete, which would likely deteriorate the size distribution after NP formation. We also propose a mechanism of the electron-induced reactions of copper(II) oxalate leading to the removal of oxalate and to the reduction of Cu^2+^ ions to elemental Cu.

## Experimental

### Preparation of copper(II) oxalate surface layers

Using a layer-by-layer approach described in detail previously [26], copper(II) oxalate was grown on carboxylic acid-terminated self-assembled monolayers (SAMs) deposited on Au surfaces. Briefly, the SAMs were prepared using a 1 mM ethanolic solution of 11-mercaptoundecanoic acid (MUA) and an incubation time of 72 h. The following layer-by-layer deposition of copper(II) oxalate was carried out by alternately dipping the substrates in ethanolic solutions of copper(II) acetate monohydrate (1 mM) and oxalic acid dihydrate (0.1 mM) for 30 and 60 min, respectively. The number of such dipping cycles was varied between 4 and 16 to generate layers with different thickness and, consequently, metal content.

### Reflection absorption infrared spectroscopy

Reflection absorption infrared (RAIR) spectra between 4000 and 500 cm^−1^ were recorded using an evacuated FTIR spectrometer (IFS 66v/S, Bruker Optics GmbH) by accumulating 400 scans. The spectrometer was equipped with a grazing incidence reflection unit and a liquid nitrogen-cooled MCT detector with sensitivity range extending down to 750 cm^−1^. The resolution was set to 4 cm^−1^ and the aperture to 1.5 mm. The chamber pressure was 7 mbar. The system was purged with N_2_ to eliminate water vapor and carbon dioxide. Background spectra were recorded on a MUA SAM.

### X-ray photoelectron spectroscopy

XPS was conducted at the Bielefeld University in a multi-technique UHV instrument (Multiprobe, Omicron Nanotechnology). Samples were stored under argon atmosphere in sealed petri dishes before being introduced to the UHV chamber. All measurements were performed using a monochromatized Al Kα X-ray source (1486.7 eV, 255 W) and a hemispherical electron energy analyzer (Omicron, Sphera). Photoelectrons were detected under an angle of 13° with respect to the surface normal. Peak positions were calibrated using the Au 4f_7/2_ peak at 84.0 eV. CasaXPS was utilized to analyze the spectra, and a Shirley background subtraction procedure was employed.

### Electron irradiation

Between the acquisitions of spectra the samples investigated by RAIRS were introduced in a dedicated UHV chamber with base pressure of 1 × 10^−8^ mbar and irradiated using an electron flood-gun (FG15/40, Specs), which generates a sufficiently divergent beam to grant a uniform irradiation of the samples. Experiments were performed at electron energies of 50 or 500 eV with exposures ranging from 125 to 30000 μC/cm^2^. Samples were exposed to air between electron irradiation and the RAIRS measurements.

Samples studied by XPS were irradiated in situ with an electron flood-gun (SL1000, Omicron) thus excluding contact of the samples with air between electron irradiation and XPS measurements. Samples were uniformly exposed to 16000 μC/cm^2^ of 50 eV electrons.

### Helium ion microscopy measurements

Helium ion microscopy (HIM) employs a finely focused beam of He^+^ ions with a diameter down to 0.35 nm, which is scanned over the sample. The secondary electrons (SE) generated by the ion impact are detected. HIM was performed with a Carl Zeiss Orion Plus®. The helium ion beam was operated at acceleration voltages between 34 and 35 kV and at currents between 0.2 and 0.3 pA. The working distance was about 11 mm at a sample tilt of 30°. Secondary electrons were collected by an Everhart–Thornley detector at 500 V grid voltage. A dwell time per pixel between 30 and 100 μs without averaging as well as 1 μs with averaging 64 lines was used. The HIM micrographs were recorded with pixel sizes between 0.49 and 0.98 nm.

## Results

### Reflection absorption infrared spectroscopy

**Figure 1:**
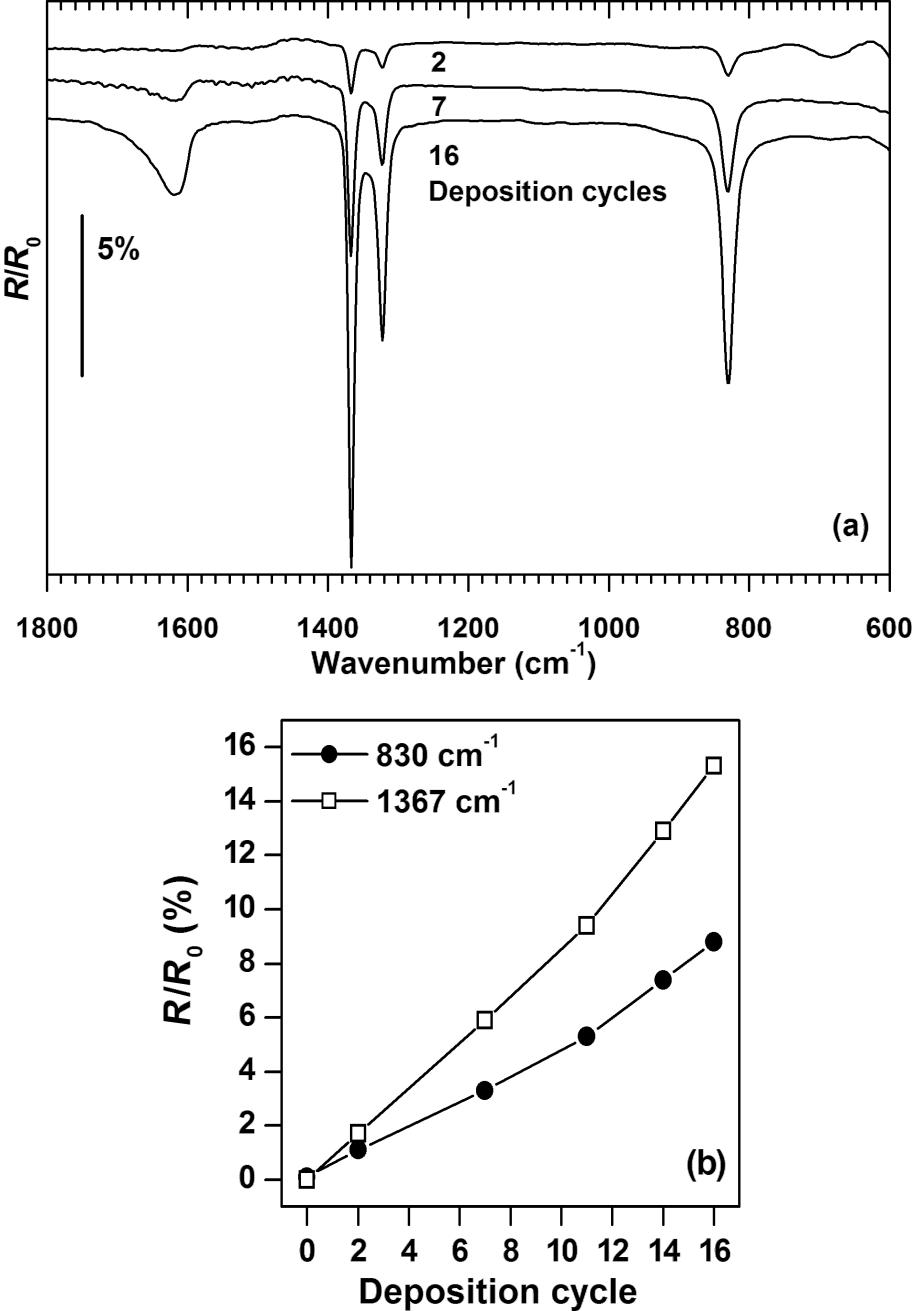
(a) Representative RAIR spectra of surface-grown copper(II) oxalate prepared by the indicated numbers of deposition cycles with acquisition being performed after dipping in oxalic acid solution (b) Band intensities as a function of the deposition cycles. All samples were grown on MUA-coated gold substrates, which were also used for recording background spectra.

As described previously, the deposition of copper(II) oxalate using a step-by-step approach of alternating dipping steps in ethanolic solutions of copper(II) acetate monohydrate and oxalic acid dihydrate can be monitored by RAIRS [26]. In close agreement with earlier results [26], spectra recorded after oxalic acid dipping steps (Figure 1a) show four bands in the range between 600 and 1800 cm^−1^, which can be assigned to characteristic vibrations of the oxalate anions. The broad band at 1620 cm^−1^ and the band at 830 cm^−1^ are uniquely assigned to the asymmetric CO_2_ stretching vibration (ν_9_, b_2u_ in Herzberg notation [27]) and to the asymmetric CO_2_ deformation (ν_12_, b_3u_ [27]). An assignment to the symmetric stretching mode of the carboxylic group has been suggested for the two sharp bands between 1400 and 1300 cm^−1^ [26]. In analogy to the band splitting described earlier for monomeric oxalate complexes [28–29], the band located at 1315 cm^−1^ in potassium oxalate [30] can split in two components in copper(II) oxalate due to coupling between oxalate ligands coordinated to a common copper atom. The band intensities show a steady increase with the number of deposition cycles (Figure 1b). This behavior has been observed before during the first 10 deposition cycles [26] but continues here to higher thickness. This result thus supports that the chosen step-by-step approach allows us to prepare surface layers with well-defined amounts of metal ions over a wide range of thicknesses.

**Figure 2:**
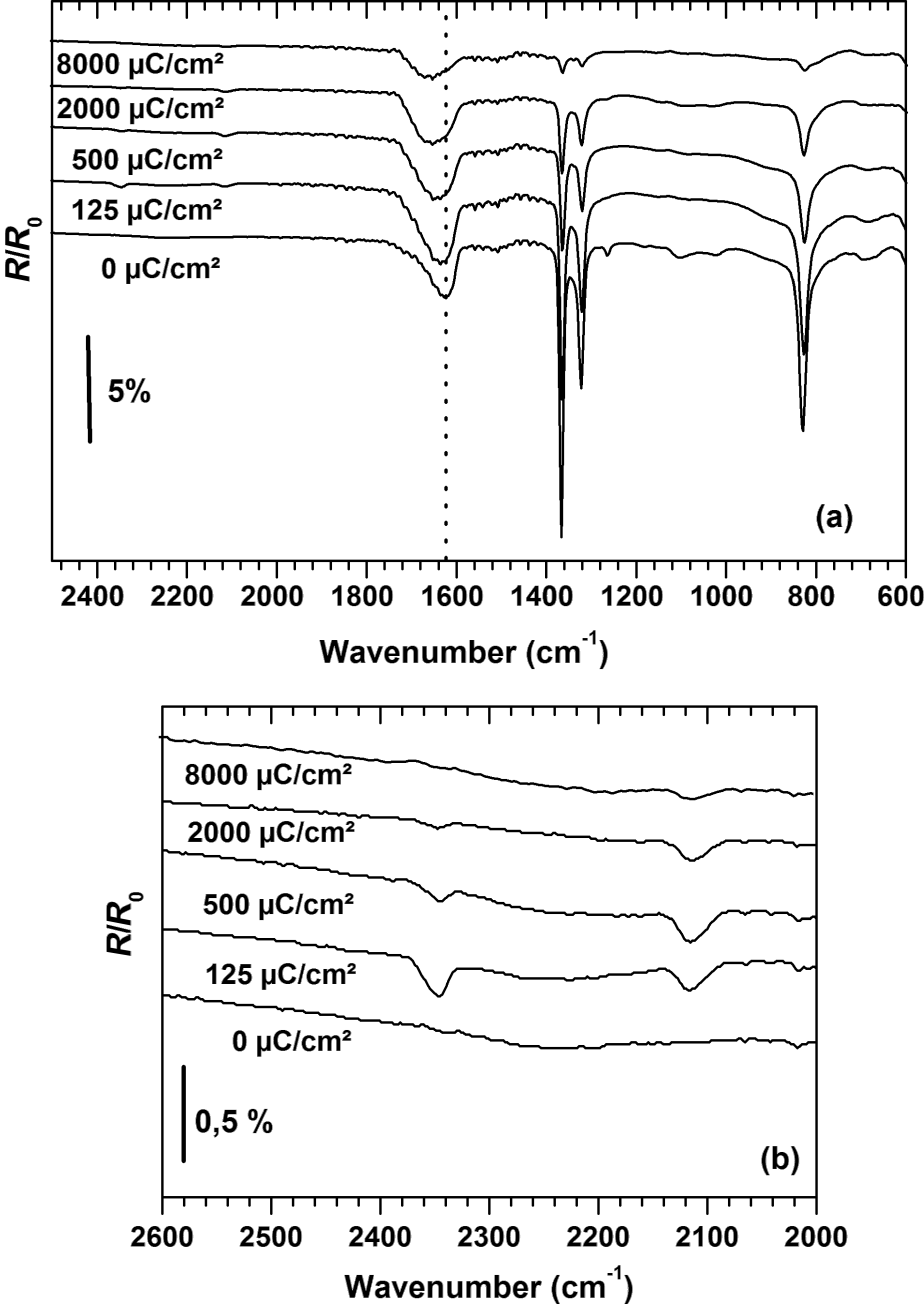
(a) RAIR spectra recorded before and after irradiation with the indicated electron exposures at 500 eV of surface grown copper(II) oxalate prepared by performing 16 deposition cycles. (b) RAIR spectra of the same samples showing the CO and CO_2_ stretching vibrational region with intensity scale magnified by a factor of 10. The samples were grown on a MUA coated gold substrate, which were also used for recording background spectra.

**Figure 3:**
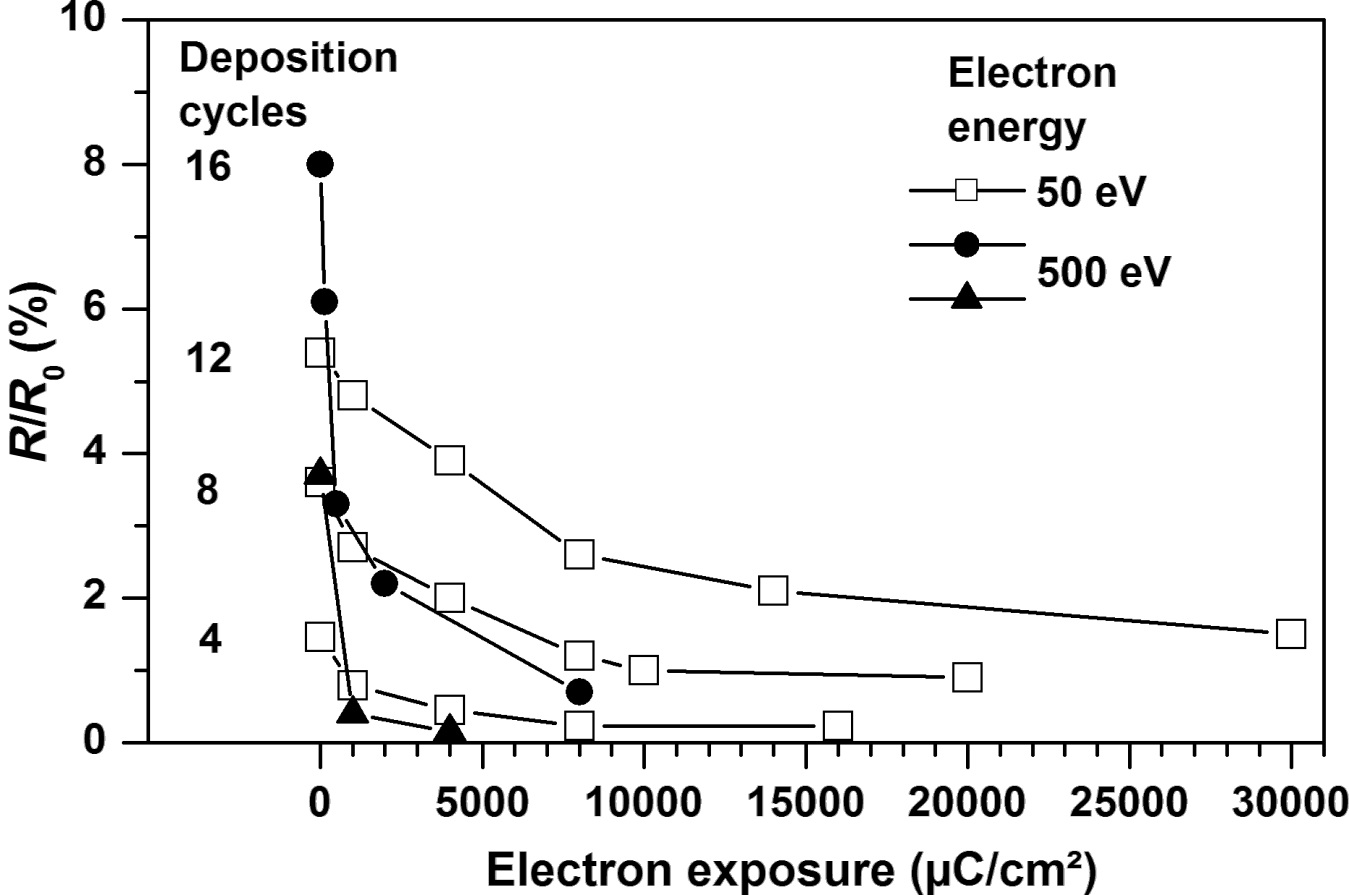
Intensity of the asymmetric CO_2_ deformation band at 830 cm^−1^ after increasing electron exposures at 50 eV (open symbols) and 500 eV (closed symbols) of surface-grown copper(II) oxalate prepared by performing different numbers of deposition cycles.

The intensity of the characteristic oxalate infrared bands, in particular below 1500 cm^−1^, decreases with increasing electron exposure, as investigated here for electron energies of 50 and 500 eV. This is in accord with a decomposition of the oxalate ions. As an example, Figure 2a shows RAIR spectra acquired after increasing exposures at 500 eV from a copper(II) oxalate surface layer prepared by 16 deposition cycles. A similar result is also obtained at lower film thickness and at 50 eV as illustrated here by plotting the intensity of the asymmetric CO_2_ deformation band at 830 cm^−1^ after increasing electron exposures (Figure 3). However, the reaction proceeds more slowly at 50 eV than at 500 eV and a complete decomposition is only achieved at sufficiently low oxalate layer thickness. More specifically, layers deposited by four deposition cycles can be fully decomposed by applying an electron exposure of 8000 μC/cm^2^ while some material is left behind after the same exposure in the case of thicker layers.

In contrast to the sharp bands below 1500 cm^−1^, the broad asymmetric CO_2_ stretching band at 1620 cm^−1^ retains a significant part of its intensity and its maximum shifts to about 1670 cm^−1^ under electron exposure (Figure 2a). However, as discussed earlier [26], the intensity of this band does not correlate directly with that of the other vibrational signals and its position and shape vary depending on the preparation conditions and water content. In fact, the bending mode of water that may be present as crystal water in copper(II) oxalate falls in the same spectral range. The shift observed here is therefore not easy to interpret but points to a change in the oxalate binding situation.

In addition to the changes described so far, new bands above 2000 cm^−1^ appear upon electron exposure at 500 eV (Figure 2b). A band at 2345 cm^−1^ can be assigned to the asymmetric stretching vibration of CO_2_ [31]. The close agreement with the frequency of 2343 cm^−1^ reported for solid CO_2_ [32] indicates a physisorbed nature of the compound. As the samples were irradiated and handled at room temperature, CO_2_ must thus be trapped within the copper(II) oxalate crystal lattice. In accordance with this, the CO_2_ intensity decreases rapidly with increasing exposure, i.e., as the deposited copper(II) oxalate layers are decomposed so that the loss of intensity most likely results from evaporation of the formed CO_2_ into the vacuum chamber. A second band appearing at 2115 cm^−1^ upon electron exposure points to the formation of chemisorbed CO with the value being characteristic for a copper surface [33]. We note that formation and retention of CO_2_ was equally observed at 50 eV but CO was not as prominent (see Supporting Information File 1, Figure S1 and Figure S2).

### Helium ion microscopy

**Figure 4:**
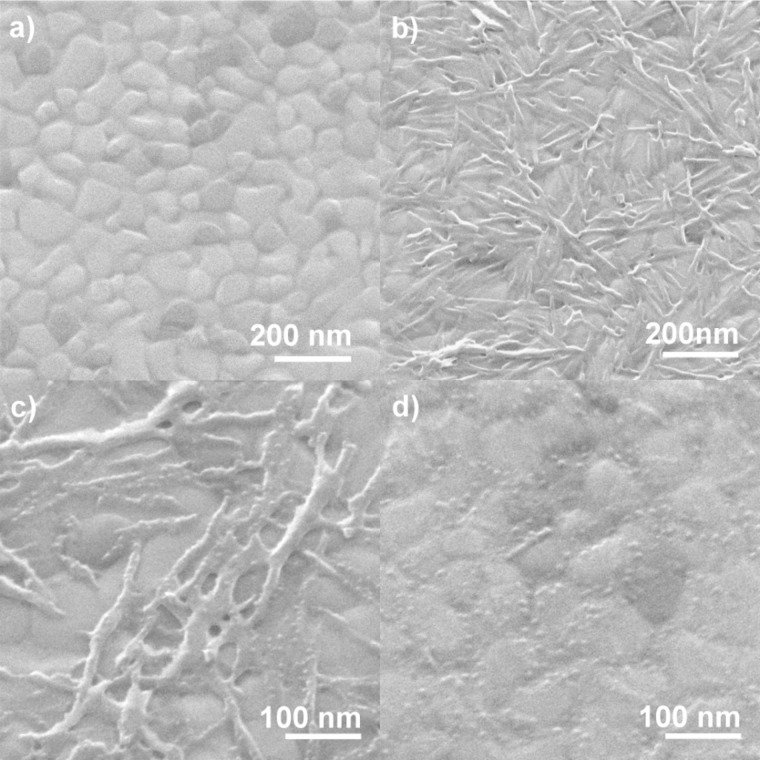
HIM images of samples after different steps of preparation and electron exposure. (a) Au substrate covered with carboxy-terminated SAM, (b) after growing on the SAM copper(II) oxalate by 16 deposition cycles, and analogous layers after electron exposure of (c) 2000 μC/cm^2^ and (d) 8000 μC/cm² at 500 eV.

**Figure 5:**
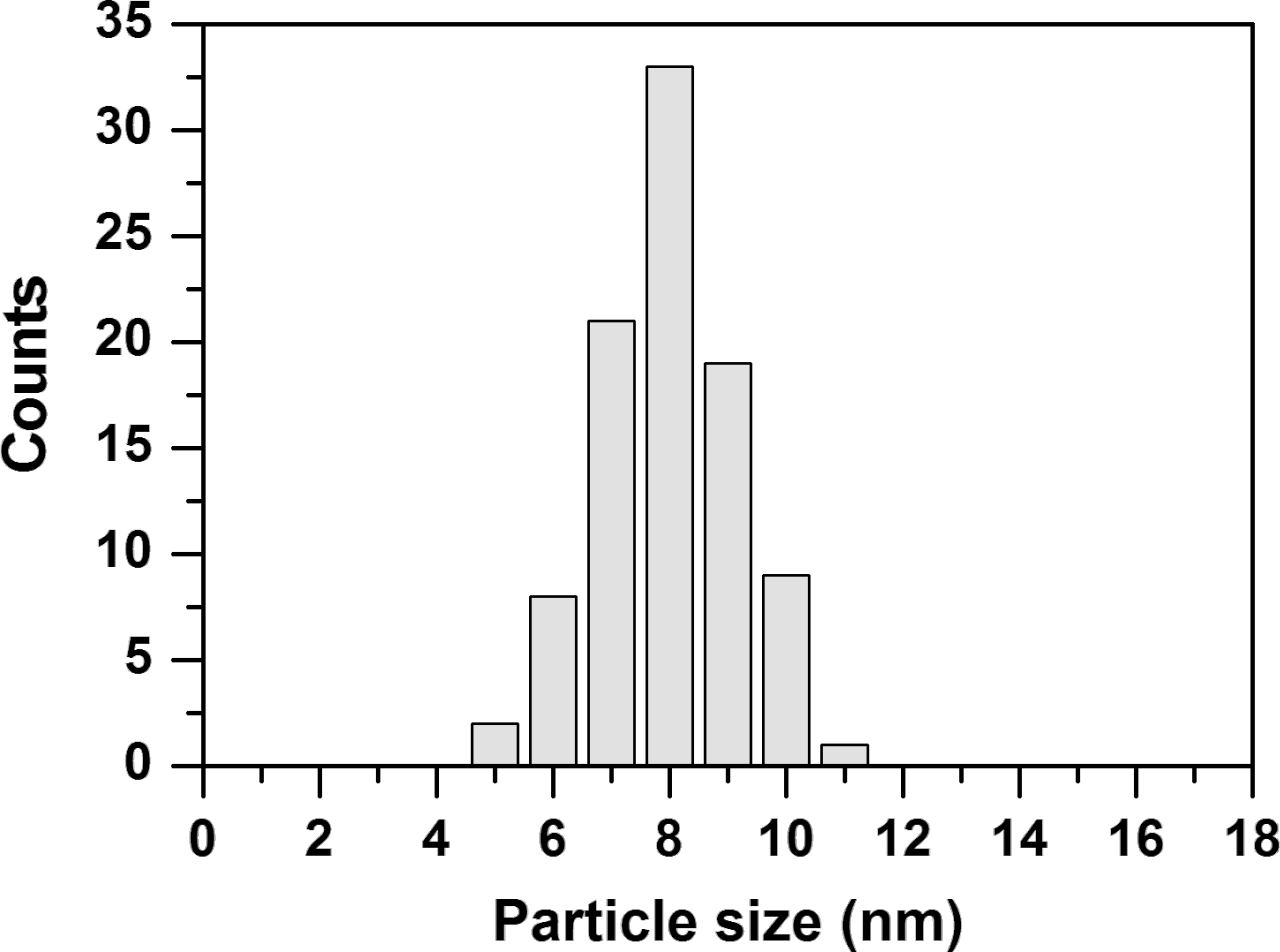
Size distribution of nanoparticles formed from surface-grown copper(II) oxalate after an electron exposure of 8000 μC/cm^2^ at 500 eV. The copper(II) oxalate was prepared by performing 16 deposition cycles. The data have been obtained by measuring the diameter of 90 particles from three different positions.

The morphology of the surface-grown copper(II) oxalate layers was investigated by using HIM. Figure 4 shows a set of images that visualizes the structural changes of the surface during deposition of copper(II) oxalate and subsequent electron exposure. In the first step, the Au-coated substrates were covered by a MUA SAM (Figure 4a). The grain boundaries between the gold crystallites are clearly visible and the facets are smooth and free of apparent defects. An image of a sample covered with copper(II) oxalate after 16 deposition cycles (Figure 4b) reveals that the material grows as needle-like structures, which are oriented parallel to the surface. In accordance with previous results [26], a closer inspection of all recorded images shows that the needles preferentially grow along the domain boundaries of the underlying gold substrate, which act as nucleation sites. After an electron exposure of 2000 μC/cm^2^ at 500 eV, the initial copper(II) oxalate crystallites are still seen (Figure 4c). This is in accordance with the presence of residual oxalate bands in a RAIR spectrum of the same sample recorded prior to the HIM measurement (Figure 2). However, small particles have started to emerge from the copper(II) oxalate needles and become even more visible after an electron exposure of 8000 μC/cm^2^ (Figure 4d). After this exposure, most of the needle-like structures have disappeared pointing to the removal of copper(II) oxalate under electron exposure. Instead, the surface is now covered with spherical nanoparticles with an average size of 8.0 ± 1.1 nm (Figure 5). Additional experiments with 16 deposited copper(II) oxalate layers and electron exposures of 16000 μC/cm^2^ produced particles with similar size distribution compared to lower exposure (see Supporting Information File 1, Figure S3). Furthermore, no effect in terms of particle quantity was observed.

**Figure 6:**
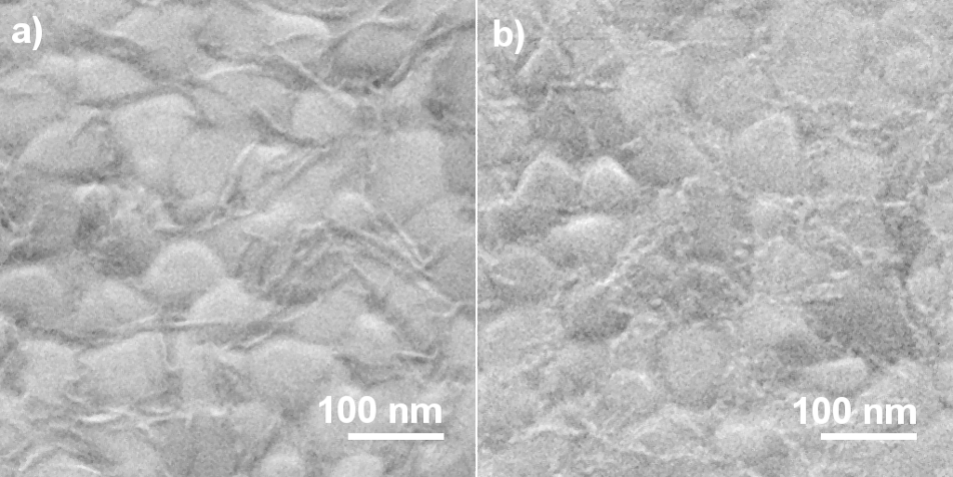
HIM images of samples after different steps of preparation and electron exposure. (a) Au substrate covered first with carboxy-terminated SAM and then with copper(II) oxalate prepared by four deposition cycles and (b) an analogous sample after electron exposure of 16000 μC/cm^2^ at 50 eV.

HIM images were also acquired of copper(II) oxalate samples prepared by four deposition cycles. An image of the pristine sample (Figure 6a) reveals again needle-like structures, which are, however, thinner and present at lower surface coverage than those obtained after 16 deposition cycles. After an electron exposure of 16000 μC/cm^2^ the needles have transformed into rows of nanoparticles (Figure 6b). This is in accordance with the RAIRS results that revealed a decay of the oxalate bands within about half of this exposure (Figure 3). Overall, HIM thus confirms that copper(II) oxalate is decomposed under electron exposure and yields a nanoparticulate material with relatively narrow size distribution.

### X-ray photoelectron spectroscopy

**Figure 7:**
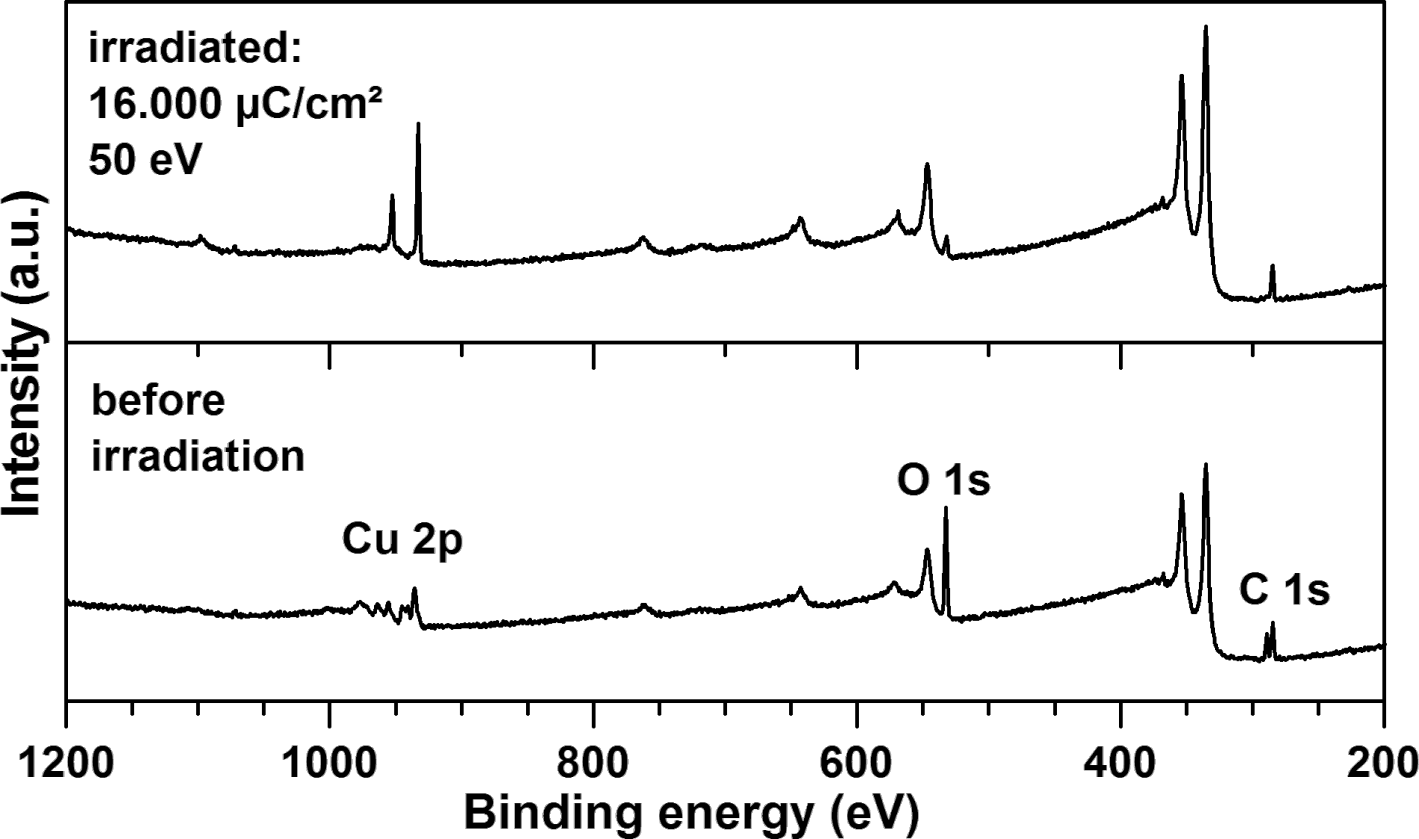
XP survey spectra recorded (a) before and (b) after an electron exposure of 16000 μC/cm^2^ at 50 eV of surface-grown copper(II) oxalate prepared by performing four deposition cycles. The sample was grown on a MUA-coated gold substrate.

In order to elucidate the nature of the nanoparticles observed by HIM and obtain further evidence for the decomposition and removal of the oxalate ions, XPS measurements were performed in combination with an in situ irradiation. As an energy of 50 eV is routinely applied in the XPS setup, copper(II) oxalate layers prepared by four deposition cycles were used which, according to the results from RAIRS, can be completely decomposed. Survey spectra (Figure 7) show that, consistent with a loss of the oxalate linker, the O 1s and C 1s signals in fact decrease strongly upon electron exposure. Furthermore the copper signals reveal a change in chemical state. High resolution spectra of the element-specific spectral ranges were thus recorded for a detailed analysis.

**Figure 8:**
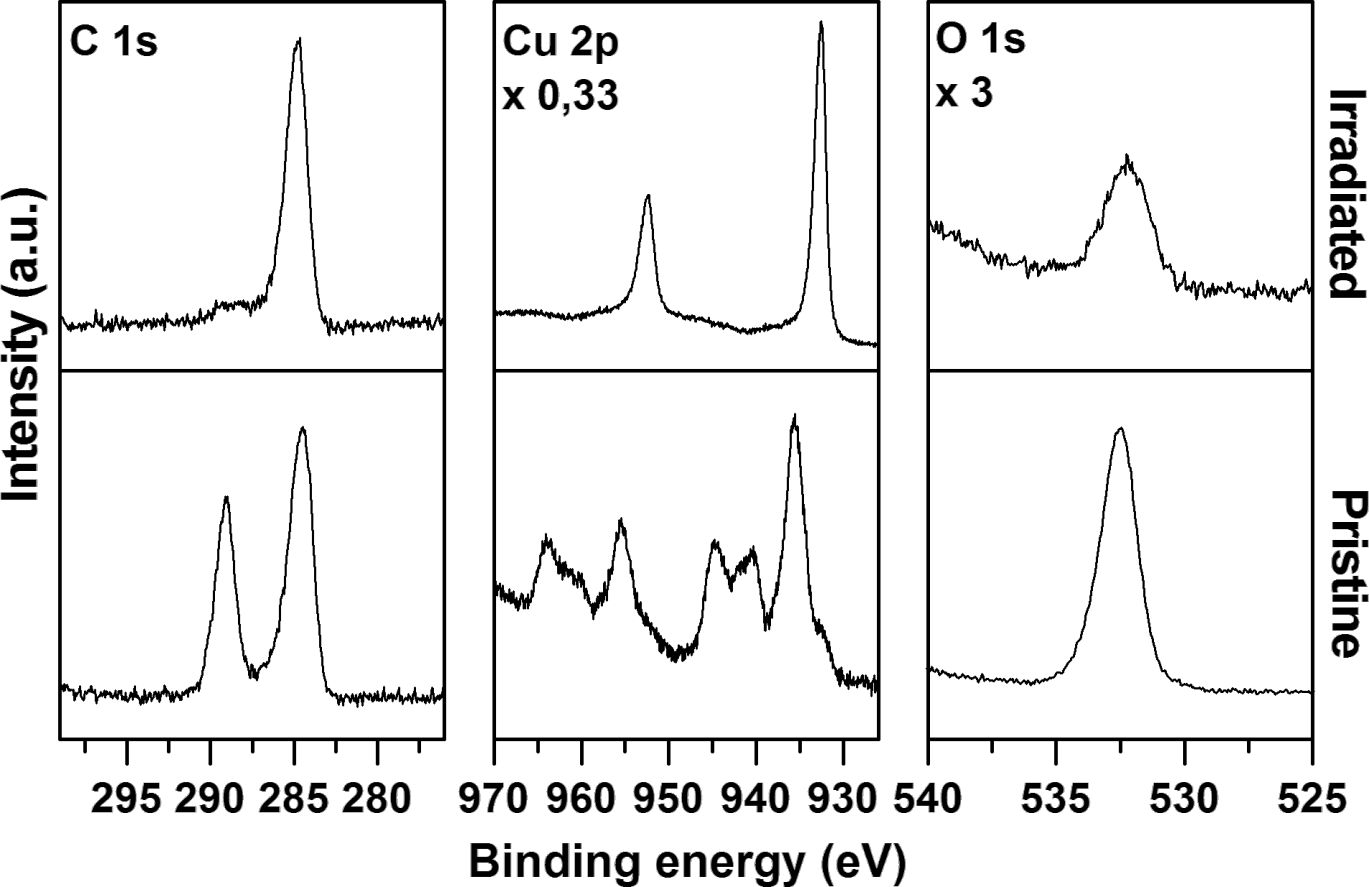
XP spectra in the ranges Cu 2p, O 1s, and C 1s recorded before (pristine) and after (irradiated) an electron exposure of 16000 μC/cm^2^ at 50 eV of surface grown copper(II) oxalate prepared by performing four deposition cycles. The sample was grown on a MUA-coated gold substrate.

Figure 8 shows the XPS spectra in the Cu 2p, O 1s, and C 1s ranges recorded prior to and after electron exposure. The XPS data are summarized and compared to literature values in Table 1. The as-prepared sample reveals two C 1s peaks at 284.5 eV, attributed to the aliphatic carbon chain of the underlying MUA SAM, and at 289.1 eV characteristic of carboxylic carbon [34] and thus assigned to the oxalate linker and to minor contributions of the MUA SAM. After irradiation, the peak at 289.1 eV nearly disappears confirming the decomposition of the oxalate linker and possibly also the decomposition of the terminal group of the underlying MUA SAM. The other signal shows a minor shift to a higher binding energy, which may relate to the overlap of the original signal with some amount of alcoholic or ether-type molecular units [35] formed by oxidation of the alkane chains in the underlying SAM.

**Table 1:** XPS data obtained before and after in situ electron exposure of 16000 μC/cm^2^ at 50 eV of a surface grown copper(II) oxalate prepared by performing four deposition cycles and comparison with literature data.

signal	binding energy (eV)		literature data (eV)	ref.
	as prepared	after exposure		

C 1s	284.5	284.8	284.7 CH_2_ aliphatic 285.0 CH_2_ aliphatic	[34] [53]
	289.1		289.6 –COOH 289.1 –COOH	[34] [53]
Cu 2p_3/2_	935.5	932.6	935.6 CuC_2_O_4_∙0.5H_2_O 932.5 metallic nanoparticle 932.6 Cu(0) 932.2 Cu_2_O 933.8 CuO	[37] [39] [38] [38] [38]
Cu 2p_1/2_	955.5	952.4	952.3 metallic nanoparticle 952.35 Cu sheet	[39] [54]
O 1s	532.5	532.2	532.5 CuC_2_O_4_∙0.5H_2_O 530.2^a^/531.6^b^ Cu_2_O 529.7^a^/531.0^b^ CuO 531.2^b^ Cu(OH)_2_	[37] [38] [38] [38]

^a^lattice O 1s; ^b^O 1s hydroxide, hydrated or defective oxygen, organic oxygen.

The Cu 2p and O 1s signals confirm the decomposition of copper(II) oxalate. Prior to electron exposure the Cu 2p spectral range shows two peaks with maxima at 935.5 and 955.5 eV, which can be attributed to the Cu 2p_3/2_ and Cu 2p_1/2_ signals, respectively. In addition, shake-up peaks located about 5 and 9.2 eV above the main 2p_3/2_ and 2p_1/2_ peaks imply the presence of Cu^2+^ [36]. The corresponding O 1s spectrum only shows a single peak at 532.5 eV. The peak positions of Cu 2p_3/2_ and O 1s are in good agreement with literature data for CuC_2_O_4_·0.5H_2_O [37].

After irradiation the copper signals have changed significantly. The shake-up peaks have disappeared, indicating a reduction of the copper(II) precursor [36,38], while the remaining signals shift to higher binding energies. The new value of the 2p_3/2_ peak agrees well with literature data for metallic copper [38] and copper nanoparticles [39]. In case of a partial reduction to Cu(I), the peak maximum would be expected at slightly lower energies (Table 1). The O 1s signal has decreased by a factor of 8 during electron exposure and shifted slightly to 532.2 eV. The remaining peak can be attributed to remaining oxalate or MUA. Formation of copper oxides is excluded from the lack of additional signals at lower binding energies (see literature values in Table 1). We thus conclude that most of the copper(II) oxalate is reduced to metallic particles during the applied electron exposure of 16000 μC/cm^2^ at 50 eV. We note, however, that XPS data acquired on samples that were exposed to air after electron irradiation revealed the presence of oxidized copper again (see Supporting Information File 1, Figure S4).

## Discussion

The combined evidence from RAIRS, HIM, and XPS shows that surface-grown copper(II) oxalate is efficiently decomposed by electron irradiation. This process is faster at 500 eV than at 50 eV, which relates to the fact that higher energy electrons (i) undergo more inelastic scattering events and thus transfer more energy to the sample and (ii) produce more secondary electrons that also play a significant role in electron-induced chemistry [40]. The RAIRS results further reveal that only sufficiently thin layers are fully decomposed in accord with a limited mean free path of electrons in a solid material [41]. The faster degradation of copper(II) oxalate at 500 eV thus also relates to the ability of these electrons to penetrate deeper in the material than 50 eV electrons [42].

HIM also reveals that degradation of copper(II) oxalate yields a nanoparticulate material with relatively narrow size distribution as compared to similar techniques. The advantage of the process is that no thermal post processing is required. As an example, gold nanoparticles generated by electron irradiation of hydrogen tetrachloroaurat (HAuCl_4_) embedded in PDDA (poly(diallyldimethyl ammonium chloride)) tends to ripen during the postpyrolysis and thus form particles with a standard deviation of size of up to 30 percent of the particle size [15].

According to XPS, the generated nanoparticles consist of metallic copper and are, however and expectedly, sensitive towards oxidation when handled in air. The particle sizes do not change upon further irradiation after complete degradation of the copper(II) oxalate. This indicates that particle growth is limited by the supply of precursor material. The relatively low electron energies applied to the samples thus do not lead to further change of the particle sizes by Ostwald ripening.

**Scheme 1:**
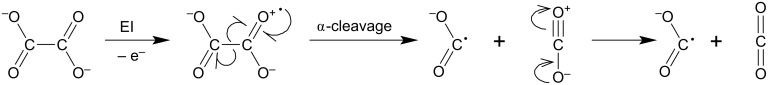
Proposed mechanism for the electron-induced decomposition of the oxalate ion.

RAIRS reveals that the decomposition of oxalate ions under electron exposure is accompanied by the formation of CO_2_ and CO. Both compounds have also been observed before as products of the electron-induced fragmentation of carboxylic acids with CO_2_ being dominant [43]. The reaction proceeds via both, dissociative electron attachment at electron energies around 1 eV, which are typical for secondary electrons and, with higher yield, above the ionization threshold. C–C bond cleavage has also been observed in mass spectra of oxalic acid as deduced from the appearance of the fragments CO_2_^+^, CO_2_H^+^ and CO_2_H_2_^+^ [44]. As electron energies above the ionization threshold have also been applied in the present study and it is difficult to conceive ionization from the copper ions, we propose that the decomposition of copper(II) oxalate is initiated by ionization of the oxalate ion and subsequent C–C bond cleavage (α-cleavage) yielding CO_2_ and a CO_2_ radical anion (Scheme 1).

A vibrational frequency of 1665 cm^−1^ has been observed for the radical anion CO_2_^−•^ [45]. This is close to the position to which the 1620 cm^−1^ band of copper(II) oxalate shifts during electron exposure. While it is tempting to assign this new band to CO_2_^−•^ regarding the mechanism proposed above, such an assignment can be ruled out here because this species is highly unstable. With a predicted reduction potential between −1.98 and −1.10 V versus NHE [46] CO_2_^−•^ is very likely to reduce adjacent copper(II) ions by decaying to more CO_2_. This must yield copper(I) ions. The reduction step required for the formation of metallic copper then very likely involves recombination of the latter ions with thermalized electrons or low-energy secondary electrons released during the initial ionization event. Altogether, this mechanism provides a reasonable scenario regarding the formation of metallic copper. We note that a band at 1650 cm^−1^ has been observed in RAIR spectra of oxalic acid adsorbed on Cu(110) and was assigned to a singly protonated species [47]. In the present experiments, protons can be supplied from the underlying MUA SAM so that the same assignment can likely be made here.

Concerning the formation of CO that is observed as chemisorbed species, two possibilities arise. The first is a dissociative adsorption of CO_2_ on the copper nanoparticles that are formed under electron exposure. This process is important for the chemical understanding of the industrial methanol synthesis with Cu–ZnO–Al_2_O_3_ catalysts [48] and has been observed on different flat and stepped copper surfaces [49–50]. On the other hand, it is known that CO is also produced by low-energy electron-induced decomposition of CO_2_ [51]. The finding that less CO is produced during electron irradiation at 50 eV than at 500 eV can then be traced back to the lower number of secondary electrons released in the first case. This also points to a significant contribution of electron-induced chemistry to the formation of CO which can be detected in RAIRS as adsorbate on the emerging copper nanoparticles.

## Conclusion

This study confirms the previous finding [26] that layer-by-layer deposition of copper(II) oxalate by alternately dipping a carboxy-terminated surface into solutions of copper(II) acetate and oxalic acid is a robust process yielding a reproducible surface coating. As an extension of this work, the electron-induced decomposition of copper(II) oxalate and the consequent formation of a nanoparticulate material is investigated here. HIM measurements of an irradiated sample of copper(II) oxalate produced by 16 deposition cycles reveals the formation of spherical nanoparticles with well-defined sizes. These particles consist of metallic copper according to XPS and their formation is accompanied by a complete degradation of the oxalate ions for which a mechanism is proposed here. Overall, the results show that copper(II) oxalate is a favorable material for the electron-induced formation of metallic copper nanoparticles on surfaces with little carbon contamination. The reduction of the material under high-vacuum conditions also offers the perspective of adding capping layers in situ via an electron-beam induced deposition process from the gas phase [1] thus addressing the problem of Cu oxidation [52].

## Supporting Information

File 1 Additional experimental data.

## References

[1] Utke I, Hoffmann P, Melngailis J (2008). J Vac Sci Technol, B.

[2] van Dorp W F, van Someren B, Hagen C W, Kruit P, Crozier P A (2005). Nano Lett.

[3] Huth M, Porrati F, Schwalb C, Winhold M, Sachser R, Dukic M, Adams J, Fantner G (2012). Beilstein J Nanotechnol.

[4] Spencer J A, Rosenberg S G, Barclay M, Wu Y-C, McElwee-White L, Howard Fairbrother D (2014). Appl Phys A.

[5] Halka V, Schmid M J, Avrutskiy V, Ma X, Schuster R (2011). Angew Chem, Int Ed.

[6] Donev E U, Hastings J T (2009). Nano Lett.

[7] Liu Y, Chen X, Noh K W, Dillon S J (2012). Nanotechnology.

[8] Bresin M, Chamberlain A, Donev E U, Samantaray C B, Schardien G S, Hastings J T (2013). Angew Chem, Int Ed.

[9] Gölzhäuser A, Geyer W, Stadler V, Eck W, Grunze M, Edinger K, Weimann T, Hinze P (2000). J Vac Sci Technol, B.

[10] Turchanin A, Gölzhäuser A (2012). Prog Surf Sci.

[11] Li Y, Kim Y N, Lee E J, Cai W P, Cho S O (2006). Nucl Instrum Methods Phys Res, Sect B.

[12] Herley P J, Jones W (1992). J Chem Soc, Faraday Trans.

[13] Yen M-Y, Chiu C-W, Chen F-R, Kai J-J, Lee C-Y, Chiu H-T (2004). Langmuir.

[14] Corbierre M K, Beerens J, Lennox R B (2005). Chem Mater.

[15] Kim Y N, Yoo S H, Cho S O (2009). J Phys Chem C.

[16] Zhang W, Song J, Liao W, Guan Y, Zhang Y, Zhu X X (2013). J Mater Chem C.

[17] Parent L R, Robinson D B, Cappillino P J, Hartnett R J, Abellan P, Evans J E, Browning N D, Arslan I (2014). Chem Mater.

[18] Jacobs B W, Houk R J T, Wong B M, Talin A A, Allendorf M D (2011). Nanotechnology.

[19] Kim S-E, Han Y-H, Lee B c, Lee J-C (2010). Nanotechnology.

[20] Botman A, Mulders J J L, Hagen C W (2009). Nanotechnology.

[21] Love J C, Estroff L A, Kriebel J K, Nuzzo R G, Whitesides G M (2005). Chem Rev.

[22] Kind M, Wöll C (2009). Prog Surf Sci.

[23] Arslan H K, Shekhah O, Wohlgemuth J, Franzreb M, Fischer R A, Wöll C (2011). Adv Funct Mater.

[24] Zacher D, Shekhah O, Wöll C, Fischer R A (2009). Chem Soc Rev.

[25] Stavila V, Volponi J, Katzenmeyer A M, Dixon M C, Allendorf M D (2012). Chem Sci.

[26] Schrader I, Wittig L, Richter K, Vieker H, Beyer A, Gölzhäuser A, Hartwig A, Swiderek P (2014). Langmuir.

[27] Paul J, Williams G P, Hoffmann F M (2003). Surf Sci.

[28] Scott K L, Wieghardt K, Sykes A G (1973). Inorg Chem.

[29] Fujita J, Martell A E, Nakamoto K (1962). J Chem Phys.

[30] Clark R J H, Firth S (2002). Spectrochim Acta, Part A: Mol Biomol Spectrosc.

[31] Millar G J, Seakins J, Metson J B, Bowmaker G A, Cooney R P (1994). J Chem Soc, Chem Commun.

[32] Yamada H, Person W B (1964). J Chem Phys.

[33] Pritchard J, Catterick T, Gupta R K (1975). Surf Sci.

[34] Cecchet F, Pilling M, Hevesi L, Schergna S, Wong J K Y, Clarkson G J, Leigh D A, Rudolf P (2003). J Phys Chem B.

[35] Seshadri K, Froyd K, Parikh A N, Allara D L, Lercel M J, Craighead H G (1996). J Phys Chem.

[36] Poulston S, Parlett P M, Stone P, Bowker M (1996). Surf Interface Anal.

[37] Nickolov R N, Donkova B V, Milenova K I, Mehandjiev D R (2006). Adsorpt Sci Technol.

[38] Biesinger M C, Payne B P, Grosvenor A P, Lau L W M, Gerson A R, Smart R S C (2010). Appl Surf Sci.

[39] Chen H, Lee J-H, Kim Y-H, Shin D-W, Park S-C, Meng X, Yoo J-B (2010). J Nanosci Nanotechnol.

[40] Böhler E, Warneke J, Swiderek P (2013). Chem Soc Rev.

[41] Seah M P, Dench W A (1979). Surf Interface Anal.

[42] Swiderek P, Jolondz E, Bredehöft J H, Borrmann T, Dölle C, Ott M, Schmüser C, Hartwig A, Danilov V, Wagner H-E (2012). Macromol Mater Eng.

[43] Houplin J, Amiaud L, Humblot V, Martin I, Matar E, Azria R, Pradier C-M, Lafosse A (2013). Phys Chem Chem Phys.

[44] Stein S E, Linstrom P J, Mallard W G NIST Mass Spec Data Center. NIST Chemistry WebBook, NIST Standard Reference Database Number 69.

[45] Hartman K O, Hisatsune I C (1966). J Chem Phys.

[46] Koppenol W H, Rush J D (1987). J Phys Chem.

[47] Martin D S, Cole R J, Haq S (2003). Surf Sci.

[48] Behrens M, Studt F, Kasatkin I, Kühl S, Hävecker M, Abild-Pedersen F, Zander S, Girgsdies F, Kurr P, Kniep B-L (2012). Science.

[49] Bönicke I A, Kirstein W, Thieme F (1994). Surf Sci.

[50] Fu S S, Somorjai G A (1992). Surf Sci.

[51] Deschamps M C, Michaud M, Sanche L (2004). J Chem Phys.

[52] Pedersen D B, Wang S (2007). J Phys Chem C.

[53] Vogt A D, Han T, Beebe T P (1997). Langmuir.

[54] Asami K (1976). J Electron Spectrosc Relat Phenom.

