# Supplementary material for: Efficient electron-induced removal of oxalate ions and formation of copper nanoparticles from copper(II) oxalate precursor layers
Source: Beilstein J Nanotechnol. 2016 Jun 13;7:852–61. doi: 10.3762/bjnano.7.77 (PMC4979688; doi:10.3762/bjnano.7.77)
Supplement: File 1 — Additional experimental data. [file Beilstein_J_Nanotechnol-07-852-s001.pdf]

Supporting Information

for

Efficient electron-induced removal of oxalate ions and formation of copper nanoparticles from copper(II) oxalate precursor layers

Kai Rückriem[1], Sarah Grotheer[1], Henning Vieker[2], Paul Penner[2], André Beyer[2], Armin Gölzhäuser[2] and Petra Swiderek*[1]

Address: [1]University of Bremen, Institute for Applied and Physical Chemistry, Fachbereich 2 (Chemie/Biologie), Leobener Straße / NW 2, Postfach 330440, D-28334 Bremen, Germany and [2]Physics of Supramolecular Systems, Bielefeld University, D-33615 Bielefeld, Germany

Email: Petra Swiderek* - swiderek@uni-bremen.de

*Corresponding author

Additional experimental data

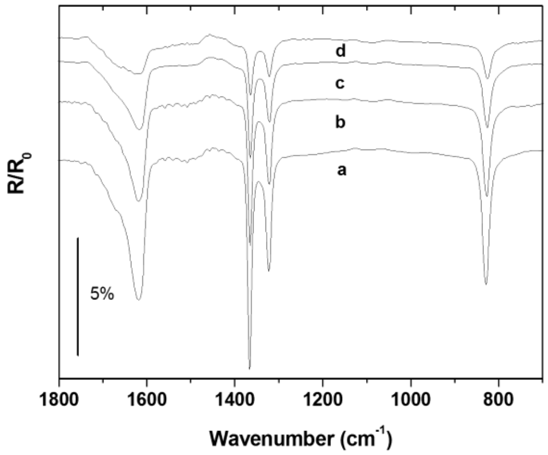

Figure S1: Additional RAIR spectra showing the electron-induced decomposition at 50 eV of surface grown copper(II) oxalate prepared by performing 12 deposition cycles. a) 0 µC/cm^2, b) 4000 µC/cm^2, c) 8000 µC/cm^2 d) 30000 µC/cm^2. The samples were grown on MUA-coated gold substrates as also used for recording background spectra.

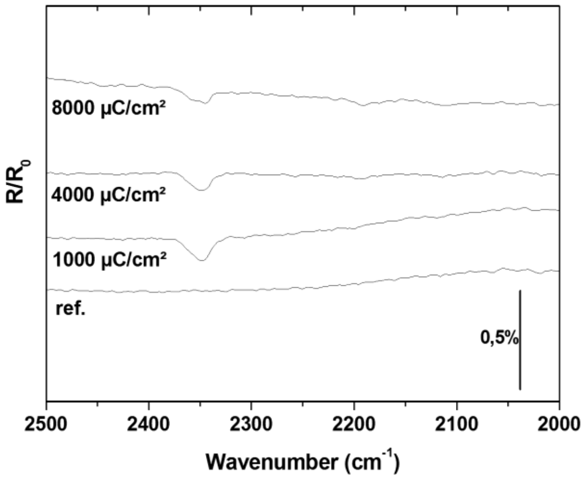

Figure S2: RAIR spectra of the CO and CO_2 stretching vibrational region recorded before and after the indicated electron exposures at 50 eV of surface-grown copper(II) oxalate prepared by performing 12 deposition cycles. The samples were grown on MUA-coated gold substrates as also used for recording background spectra.

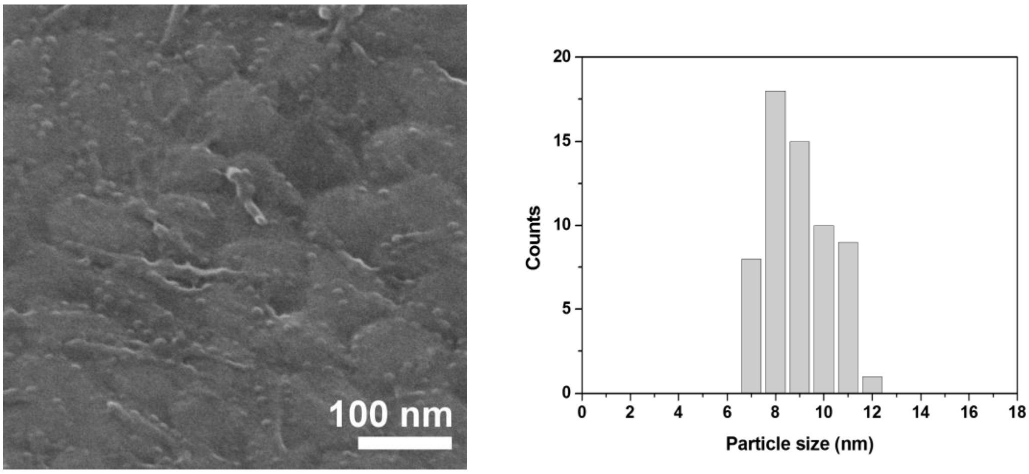

Figure S3: HIM Image and associated size distribution of a sample after growing on the SAM copper(II) oxalate by 16 deposition cycles and additional electron exposure of 16000 μC/cm^2 at 500 eV. The copper(II) oxalate was prepared on a MUA-coated Au substrate by performing 16 deposition cycles. The size distribution has been obtained by measuring the diameter of 62 particles from two different positions.

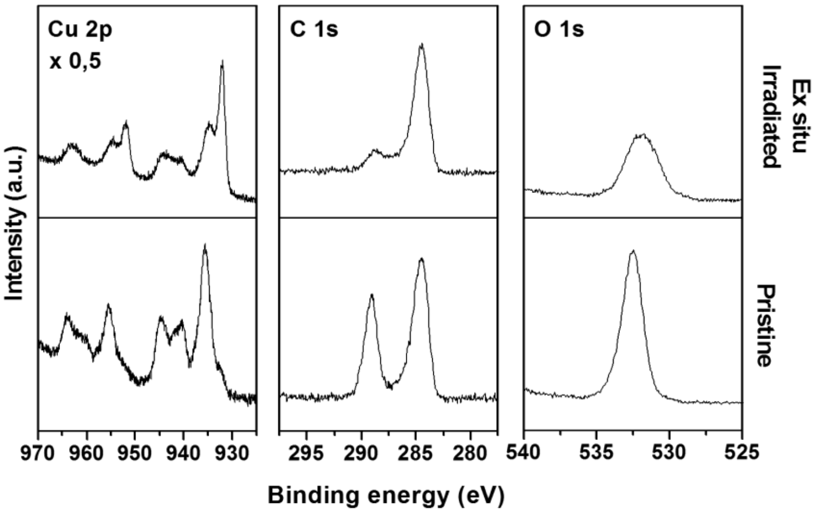

Figure S4: XPS spectra in the ranges of Cu 2p, O 1s, and C 1s recorded before (pristine) and after ex situ irradiation with an electron exposure of 16000 μC/cm^2 at 50 eV of surface-grown copper(II) oxalate prepared by performing four deposition cycles. The sample was grown on a MUA-coated gold substrate. The Cu 2p signal points to the reappearance of oxidized species following exposure to air.
